# Supplementary material for: HDAC3: A Multifaceted Modulator in Immunotherapy Sensitization
Source: Vaccines (Basel). 2025 Feb 13;13(2):182. doi: 10.3390/vaccines13020182 (PMC11860249; doi:10.3390/vaccines13020182)
Supplement: Supplementary file 1 [file vaccines-13-00182-s001.zip › vaccines-3407522-supplementary.pdf]

Table1. **Clinical trial of dual immunotherapy combination therapy with the HDAC inhibitor in the past five years**

| Trial Number | Launch | Phase | Study Status | HDAC Inhibitor | Type of Immunotherapy | Outcome | Number of Samples | Tumor type | Refs |
|---|---|---|---|---|---|---|---|---|---|
| NCT04512534 | 2020 | II | Recruiting | Chidamide | Sintilimab | - | - | Peripheral T Cell Lymphoma | [1] |
| NCT04514081 | 2020 | II | Recruiting | Chidamide | Decitabine+Camrelizumab | - | - | Classical Hodgkin Lymphoma | [2] |
| NCT05320640 | 2022 | I/II | Recruiting | Chidamide | Decitabine + anti-PD1/PD-L1/CTLA4 antibodies | - | - | Relapsed/Refractory Non-Hodgkin Lymphoma and Advanced Solid Tumors | [3] |

| | | | | | | | | | |
|---|---|---|---|---|---|---|---|---|---|
| NCT04651127 | 2020 | Ib/II | Unknown status | Chidamide | Toripalimab | - | - | Metastatic, persistent, or recurrent cervical | [4] |
| NCT04233294 | 2020 | II | Recruiting | Chidamide | Decitabine + Camrelizumab | Chidamide combined with Decitabine and Camrelizumab (CDP) was well tolerated among 52 patients, demonstrating an objective response rate of 94% (95% confidence interval [CI], 84-99). Among these, 50% of patients (95% CI, 36-64) achieved a complete response (CR). All patients who had previously failed to respond to Decitabine and Camrelizumab treatment exhibited a therapeutic response following CDP treatment. CDP treatment appears to promote the activation of multiple tumor-reactive CD8+ T cells while inhibiting the proliferation of IL21+CD4+ T cells through the inhibition of STAT1/3 signaling, thereby mitigating their immunosuppressive effects | - | Classical Hodgkin Lymphoma | [5] |

| | | | | | | | | | |
|---|---|---|---|---|---|---|---|---|---|
| NCT05113355 | 2021 | II | Unknown status | Chidamide | Sintilimab | - | - | Chemotherapy-refractory Advanced High-grade Neuroendocrine N | [6] |
| NCT04296942 | 2021 | I | Terminated | Entinostat | BN-Brachyury+ Adotrastuzumab Emtansine+M7824 | One participant was accrued, and the study was stopped due to new safety data from the company for M7824 and slow accrual | - | Advanced Stage Breast Cancer | [7] |
| NCT04553393 | 2020 | I/II | Unknown status | Chidamide | Decitabine-primed Tandem CAR19/20 engineered T cells+ Decitabine | - | - | Relapsed and/or Refractory B cell Non-Hodgkin's Lymphoma with hugh tumor burden | [8] |
| NCT05694936 | 2023 | II | Recruiting | Sodium Valproate | Anti-EGFR monoclonal antibody (panitumumab or cetuximab) | - | - | RAS wild type metastatic colorectal cancer | [9] |

| | | | | | | | | | |
|---|---|---|---|---|---|---|---|---|---|
| NCT05154994 | 2022 | I | Suspended | belinostat | durvalumab | Pending approval of cohort expansion | - | Treat patients with urothelial cancer that has spread to other places in the body (metastatic) | [10] |
| NCT04956302 | 2021 | I | Terminated | panobinostat | Daratumumab+ bortezomib +dexamethasone | PI Decision | - | Treat multiple myeloma that has come back (relapsed) or has not responded to treatment (refractory) | [11] |
| NCT04133948 | 2020 | I/Ib | Active, not recruiting | domatinostat | Nivolumab+ipilimumab | The combination of TAL-012 and nivolumab was generally well-tolerated, with manageable side effects(details unreported). Objective responses were seen in a subset of patients, indicating potential efficacy for certain cancers. The maximum tolerated dose (MTD) and RP2D were identified, enabling further testing in later-phase studies. | - | IFN-gamma signature high patients with Unknown Primary Melanoma | [12] |

| | | | | | | | | | |
|---|---|---|---|---|---|---|---|---|---|
| NCT05438706 | 2022 | II | Unknown status | chidamide | Camrelizumab+ carboplatin/ capecitabine | - | - | Second and Third Line Treatment of Relapsed/Metastatic Triple-negative Breast Cancer | [13] |
| NCT04708470 | 2021 | I/II | Recruiting | Entinostat | Bintrafusp Alfa+PDS01ADC | - | - | Advanced Cancers (HPV-Associated Malignancies, Small Bowel, and Colon Cancers) | [14] |
| NCT04631029 | 2021 | I | Completed | Entinostat | Atezolizumab+Carboplatin + Etoposide | Three patients were enrolled and treated at DL1 with entinostat 2 mg. Patients were aged 69-83; 2 male, 1 female; 2 were ECOG 1, and 1 was ECOG 0;Addition of entinostat to atezolizumab, carboplatin, and etoposide is unsafe and resulted in hair loss and severe neutropenia, thrombocytopenia. Further exploration of entinostat with carboplatin, etoposide, and atezolizumab should not be explored | 3 | Previous Untreat Extensive-Stage Small Cell Lung Cancer | [15] |

**References**


1. Xu, Q.Y.; Yang, H.Y.; Li, M.W.; He, Z.D.; Hong, H.Y.; Peng, Z.G. Sintilimab combined with chidamide in the treatment of extranodal nature killer/T-cell lymphoma with secondary hemophagocytic lymphohistiocytosis: Two case reports and literature review. *Medicine (Baltimore).* **2022**, 101, 38, e30731. https://doi.org/10.1097/MD.0000000000030731
2. Kong, F.C.; Qi, L.; Zhou, Y.L.; Yu, M.; Huang, W.F.; Li, F. Chidamide, Decitabine, Cytarabine, Aclarubicin, and Granulocyte Colony-stimulating Factor Therapy for Patients with Relapsed/Refractory Acute Myeloid Leukemia: A Retrospective Study from a Single-Center. *Curr Med Sci.* **2023**, 43, 6, 1151-1161. https://doi.org/10.1007/s11596-023-2805-7
3. Zhang, W.; Shen, H.; Zhang, Y.; Wang, W.; Hu, S.; Zou, D.; Zhou, D. Circulating PD-1 (+) cells may participate in immune evasion in peripheral T-cell lymphoma and chidamide enhance antitumor activity of PD-1 (+) cells. *Cancer Med.* **2019**, 8, 5, 2104-2113. https://doi.org/10.1002/cam4.2097
4. Que, Y. ; Zhang, X.L. ; Liu, Z.X.; Zhao, J.J. ; Pan, Q.Z. ; Wen, X.Z.; Xiao, W.; Xu, B.S.; Hong, D.C.; Guo, T.H.; etal. Frequent amplification of HDACgenes and efficacy of HDACinhibitor chidamide andPD-1 blockade combination in softtissue sarcoma. *J Immunother Cancer*. **2021**, *9*, e001696.https://doi.org/10.1136/jitc-2020-001696
5. Nie, J.; Wang, C.; Zheng, L.; Liu, Y.; Wang, C.; Chang, Y.; Hu, Y.; Guo, B.; Pan, Y.; Yang, Q.; et al. Epigenetic agents plusanti-PD 1 reprogram thetumor microenvironment andrestore antitumor efficacy in Hodgkin lymphoma. *Blood.* **2024**, *144*, 1936-1950. https://doi.org/10.1182/blood.2024024487
6. Chen, C.; Zhang, W.; Zhou, D.; Zhang, Y.Sintilimab and Chidamide for Refractory Transformed Diffuse Large BCell Lymphoma: ACase Report and A Literature Review. *Front Oncol*. **2021**, *11*, 757403. https://doi.org/10.3389/fonc.2021.757403
7. Gatti-Mays, M.E.; Gameiro, S.R.; Ozawa, Y.; Knudson, K.M.; Hicks, K.C.; Palena, C.; Cordes, L.M.; Steinnberg, S.M.; Francis, D.; Karzai, F.; et al.Improving the Odds in Advanced Breast Cancer WithCombination Immunotherapy: Stepwise Addition of Vaccine, Immune Checkpoint Inhibitor, Chemotherapy, and HDACInhibitor in Advanced Stage Breast Cancer. *Front Oncol*. **2021**, *10*, 581801.https://doi.org/10.3389/fonc.2020.581801
8. Zhu, M.; Han,Y.; Gu, T; Wang, R.; Si, X.; Kong, D.; Zhao, P.; Wang, X.; Li, J.; Zhai, X.; et al. Class I HDAC inhibitors enhance antitumor efficacy and persistence ofCAR-T cells by activation ofthe Wnt pathway. *Cell Rep*. **2024**, *43*, 114065. https://doi.org/10.1016/j.celrep.2024.114065
9. Caponigro, F.; DiGennaro, E.; Ionna, F.; Longo, F.; Aversa, C.; Pavone, E.; Maglione, M.G.; Di Marzo, M.; Muto, P.; Cavalcanti, E.; etal. Phase II clinical study of valproic acidplus cisplatin andcetuximab in recurrent and/or metastatic squamous cell carcinoma of Head andNeck-V-CHANCE trial. *BMC Cancer*. **2016**, *16*, 918. https://doi.org/10.1186/s12885-016-2957-y
10. Gupta, S.; Albertson, D.J.; Parnell, T.J.; Butterfield, A.; Weston, A. Pappas, L.M.;, Dalley, B.; O'Shea, J.M.; Lowrance, W.T.; Cairns, B.R.; et al. Histone Deacetylase Inhibition Has Targeted Clinical Benefit inARID1A-Mutated Advanced Urothelial Carcinoma. *Mol Cancer Ther*. **2019**, *18*, 185-195. https://doi.org/10.1158/1535-7163.MCT-17-0957
11. Pan, D.; Mouhieddine, T.H.; Upadhyay, R.; Casasanta, N.; Lee, A.; Zubizarreta, N.; Moshier, E.; Richter, J. Outcomes with panobinostat in heavily pretreated multiple myeloma patients. *Semin Oncol*. **2023**, *50*, 40-48. https://doi.org/10.1053/j.seminoncol.2023.03.006
12. Gorry, C.; McCullagh, L.; O'Donnell, H.; Barrett, S.; Schmitz, S.; Barry, M.; Curtin, K.; Beausang, E.; Barry, R.; Coyne, I. Neoadjuvant treatment for stage IIIand IV cutaneous melanoma. *Cochrane Database Syst Rev*. **2023**, *1*, CD012974. https://doi.org/10.1002/14651858.CD012974.pub2

13. Xia, Y. ; Li, Q.H. ; Liu, T.; Liu, X-X.; Pan, H-X.; Zhang, L-L.; Zhu, Z. Salvage camrelizumab for anintractable NK/T celllymphoma patient withtwo instances of intestinal perforation: a case report and literature review. *Eur Rev Med Pharmacol Sci*. **2023**, *27*, 4570-4577. https://doi.org/10.26355/eurrev_202305_32463
14. Hicks, K.C.; Knudson, K.M.; Lee, K.L.; Hamilton, D.H.; Hodge, J.W.; Figg, W.D.; Ordentlich, P.; Jones, F.R.; Rabizadeh, S. Soon-Shiong, P.; etal. Cooperative Immune-Mediated Mechanisms of theHDAC Inhibitor Entinostat, an IL15 Superagonist, and a Cancer Vaccine Effectively Synergize as a Novel Cancer Therapy. *Clin Cancer Res*. **2020,** *26*, 704-716. https://doi.org/10.1158/1078-0432.CCR-19-0727
15. Gentzler, R.D. ; Villaruz, L.C. ; Rhee, J.C.; Horton, B.; Mock, J.; Hanley, M. ; Kim, K. ; Rudek, M.A. ; Phelps, M.A. ; Pierkarz, R.; et al. Phase I Study ofEntinostat, Atezolizumab, Carboplatin, and Etoposide inPreviously Untreated Extensive-Stage Small Cell LungCancer, ETCTN 10399. *Oncologist*. **2023**, *28*, 1007-e1107. https://doi.org/10.1093/oncolo/oyad221
